# Tracers for non-invasive radionuclide imaging of immune checkpoint expression in cancer

**DOI:** 10.1186/s41181-019-0078-z

**Published:** 2019-11-06

**Authors:** Peter Wierstra, Gerwin Sandker, Erik Aarntzen, Martin Gotthardt, Gosse Adema, Johan Bussink, René Raavé, Sandra Heskamp

**Affiliations:** 1 Department of Radiology and Nuclear Medicine, Radboud University Medical Center, Radboud Institute for Molecular Life Sciences, P.O. Box 9101, 6500 HB Nijmegen, The Netherlands; 2 Department of Radiation Oncology, Radboud university medical center, Radboud Institute for Molecular Life Sciences, P.O. Box 9101, 6500 HB Nijmegen, The Netherlands

**Keywords:** Immune checkpoint, Immune checkpoint imaging, Tumor expression, PET, SPECT, PD-1, PD-L1, CTLA-4, OX40, CD276, CD80, IDO1, A2aR

## Abstract

**Abstract:**

Immunotherapy with checkpoint inhibitors demonstrates impressive improvements in the treatment of several types of cancer. Unfortunately, not all patients respond to therapy while severe immune-related adverse effects are prevalent. Currently, patient stratification is based on immunotherapy marker expression through immunohistochemical analysis on biopsied material. However, expression can be heterogeneous within and between tumor lesions, amplifying the sampling limitations of biopsies. Analysis of immunotherapy target expression by non-invasive quantitative molecular imaging with PET or SPECT may overcome this issue. In this review, an overview of tracers that have been developed for preclinical and clinical imaging of key immunotherapy targets, such as programmed cell death-1, programmed cell death ligand-1, IDO1 and cytotoxic T lymphocyte-associated antigen-4 is presented. We discuss important aspects to consider when developing such tracers and outline the future perspectives of molecular imaging of immunotherapy markers.

**Graphical abstract:**

Current techniques in immune checkpoint imaging and its potential for future applications

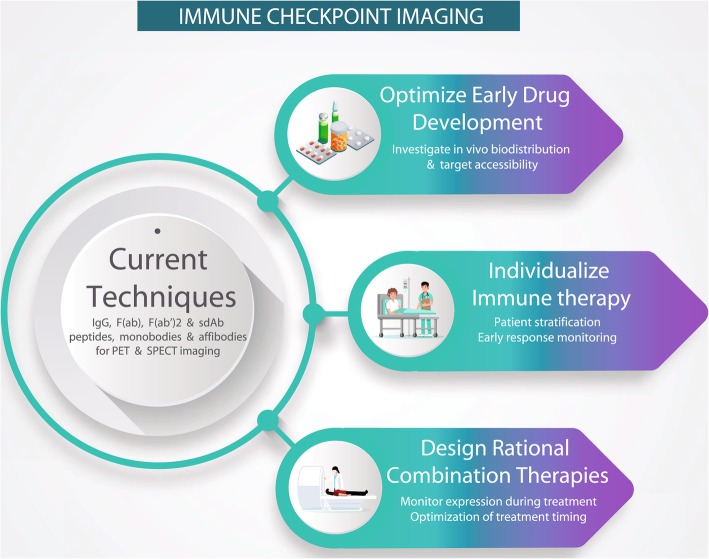

## Background

Despite a rapidly expanding therapeutic arsenal and improved understanding of its biology, cancer remains one of the major causes of mortality in the western world (Organisation WH 2011). Recent developments in cancer immunotherapy have shifted focus towards immune checkpoint inhibitors. Healthy tissues and immune cells can express cell-surface molecules to regulate the immune response and prevent auto-immune reactions, so called immune-checkpoints. Tumor cells can also (over-)express these checkpoint molecules, allowing them to escape immune surveillance (Iwai et al. 2002; Blank et al. 2005). By specifically modulating the interaction of stimulatory or inhibitory immune checkpoint molecules using monoclonal antibodies (mAb), anti-tumor immune responses can be reinvigorated and result in enhanced tumor cell recognition and killing (Zitvogel and Kroemer 2012). As a consequence of its own success, the number of clinical trials investigating new treatment regimens based on immune checkpoint inhibition (ICI) is overwhelming (Shalabi et al. 2017). However, due to a considerable group of non-responders and immune-related adverse effects associated with these therapies and considerable costs, there is a growing demand for tools that allow the use of immune therapy in the most effective way, i.e. maximizing the likelihood of response. Therefore, two strategies have been put forward; First, rational design of novel combination treatments with increased efficacy, and second, improved selection of patients who are most likely to benefit from treatment. Currently, immunohistochemical (IHC) analysis on biopsied material is the gold standard for patient therapy stratification. However, various studies have demonstrated the limitations of biopsies, namely the various sampling limitations and invasiveness of the procedure (Daud et al. 2016). Being non-invasive, sensitive, and quantitative, positron emission tomography (PET) imaging allows for longitudinal and repetitive assessment on a whole body scale of immune checkpoint expression. As such, PET imaging represents a powerful tool to fulfill these needs in oncology (Fruhwirth et al. 2018). In this review we provide a comprehensive overview of all presently published literature on radiotracers developed for immune checkpoint imaging (see Table 1).

**Table 1:** Overview of nuclear imaging tracers for immune checkpoints. Only tracers that have been published and used in at least preclinical in vivo studies are described in the tables below

Target	Name	Construct	Label	Timing	Tumor type /tissue	Therapeutic use	Reference
Clinicaly used							
PD-1	^89^Zr-Nivolumab	IgG	^89^Zr	144 h	NSCLC	Yes	(Niemeijer et al. 2018)
PD-L1	^89^Zr-Nivolumab	IgG	^89^Zr	4 and 7 d	Bladder cancer, NSCLC, or TNBC	Yes	(Bensch et al. 2018)
PD-L1	^18^F-B MS-986192	Adnectin	^18^F	Dynamic PET immediately, static acquisition after 1 h	NSLC	No	(Niemeijer et al. 2018)
IDO/TDO	Alpha-[^11^C]-methyll-tryptophan ([11C]AMT)	Small molecule	^11^C	Dynamic scan initiate during tracer infusion, to 25 min p.i.	Glioblastoma, Gliomas, meningiomas, NSCLS, breast carcinomas, 3C prostate model	Yes	(Juhasz et al. 2006, 2009, 2012; Zitron et al. 2013; Michelhaugh et al. 2017; Guastella et al. 2016)
A2aR	[^11^C]Preladenant	Small molecule	^11^C	Dynamic scan initiate during tracer infusion, to 60 min p.i.	Cerebral A2aR imaging	Yes	(Zhou et al. 2017a, 2017b, 2017c, 2017d; Sakata et al. 2017; Ishibashi et al. 2018; Zhou et al. 2014)
A2aR	[^11^C]TMSX	Small molecule	^11^C	Dynamic scan initiate during tracer infusion	Cerebral A2aR imaging, Brown Fat	Yes	(Rissanen et al. 2013; Mishina et al. 2007, 2011; Naganawa et al. 2007, 2014; Lahesmaa et al. 2018; Rissanen et al. 2015)
Preclinically used							
PD-1	^64^Cu-anti-mouse- PD-1	IgG	^64^Cu	1–48 h	B16-F10 melanoma	No	(Natarajan et al. 2017)
PD-1	^89^Zr/^64^Cu-pembrolizumab	IgG	^89^Zr, ^64^Cu	1–144 h	A375 melanoma with human peripheral blood mononuclear cells	No	(Natarajan et al. 2018a)
PD-1	^64^Cu-pembrolizumab	IgG	^64^Cu	1–48 h	293 T/hPD-1 and A375 melanoma with human peripheral blood mononuclear cells	No	(Hettich et al. 2016)
PD-1	^64^Cu-anti-mouse PD-1	IgG	^64^Cu	24 h	Naïve and PD-1^+/+^ mice, B16-F10 melanoma	No	(England et al. 2017)
PD-1	^89^Zr-pembrolizumab	IgG	^89^Zr	0.5–168 h	Human PBMCs	No	(England et al. 2018)
PD-1	^89^Zr-nivolumab	IgG	^89^Zr	3–168 h	A549 human lung cancer	No	(Bensch et al. 2018)
PD-L1	C3, C7, E2 and E4	Nanobody	^99m^Tc	1 h	TC-1 myeloma	No	(Broos et al. 2017)
PD-L1	^111^In-PD-L1.3.1	IgG	^111^In	1–7 d	MDA-MB-231, SK-Br-3, SUM149, BT474, MCF-7	No	(Heskamp et al. 2015, 2019)
PD-L1	^111^In-PD-L1-mAb	IgG	^111^In	48–120 h	MDA-MB-231, SUM149, H2444, H1155	No	(Chatterjee et al. 2017)
PD-L1	WL12	Peptide	^64^Cu	10 min-120 h	hPD-L1, CHO	No	(Chatterjee et al. 2017)
PD-L1	[^18^F]AlF-ZPD-L1_1	Affibody	^18^F	0 min	LOX, SUDHL6	No	(Gonzalez Trotter et al. 2017)
PD-L1	WL12	Peptide	^68^Ga	60 min	hPD-L1, CHO	No	(De Silva et al. 2018)
PD-L1	^18^F-BMS-986192	Adnectin	^18^F	2 h	L2987, HT-29	Yes	(Donnelly et al. 2018)
PD-L1	α-PD-L1 (10F.9G2)	IgG	^64^Cu	24 h	–	No	(England et al. 2017)
PD-L1	^18^F-B3	Single domain antibody (sdAb)	^18^F	–	–	No	(Ingram et al. 2017)
PD-L1	anti-PD-L1	IgG	^111^In	1, 24 and 72 h	NT2.5	No	(Josefsson et al. 2016)
PD-L1	^89^Zr anti-PD-L1	IgG	^89^Zr	48 and 96 h	MEER, B16F10	No	(Kikuchi et al. 2017)
PD-L1	WL12	Peptide	^64^Cu	2 h	H226, HCC827	No	(Kumar et al. 2019)
PD-L1	Atezolizumab	IgG	^64^Cu	24 and 48 h	CHO-hPD-L1, MDA-MB-231, SUM149	Yes	(Lesniak et al. 2016)
PD-L1	^89^Zr-Df-KN035	IgG	^89^Zr	24 and 120 h	LN229	Yes	(Li et al. 2018)
PD-L1	High-affinity consensus (HAC) PD-1, and derivates	Peptide	^68^Ga, ^64^Cu	1 h	CT26 and CT26^PD-L1+^	No	(Mayer et al. 2017)
PD-L1	Atezolizumab	IgG	^89^Zr	2, 24,48, 72 and 96 h	B16F10	Yes	(Moroz et al. 2018)
PD-L1	C4	IgG	^89^Zr	2, 24,48, 72 and 96 h	B16F10	No	(Natarajan et al. 2019)
PD-L1	FN3_hPD-L1_	Adnectin	^64^Cu	1–24 h	CT26, Raji, MDA-MB-231	No	(Nedrow et al. 2017a)
CTLA-4	Anti-mouse CTLA-4	IgG	^64^Cu	48 h	CT26	No	(Higashikawa et al. 2014)
CTLA-4	Ipilimumab	IgG	^64^Cu	48 h	A549 lung carcinoma xenograft	Yes	(Ehlerding et al. 2017, 2019)
CTLA-4	Ipilimumab-F (Ab’)_2_	F (Ab’)_2_	^64^Cu	48 h	Activated human T cells	No	(Ehlerding et al. 2019)
CTLA-4	H11, H11-PEG20	VHH, PEGylated VHH	^18^F, ^89^Zr	90 min and 24 h	B16F10	No	(Ingram et al. 2018)
CD80/ CD86	Belatacept	IgG1 Fc fused with CTLA-4 extracellular domain	^111^In	18–48 h	Raji	Yes	(Meletta et al. 2016)
CD80	[^11^C]AM7	Small molecule	^11^C	1 min	APCs in human atherosclerotic plaques	No	(Meletta et al. 2017)
OX40	AbOX40	Antibody	^64^Cu	2–9 days	A20	yes	(Alam et al. 2018)
IDO/TDO	[^18^F]IDO49	Small molecule	^18^F	Dynamic scan initiate during tracer infusion	HeLa xenografts	Yes	(Huang et al. 2017)
IDO/TDO	1-N-[^11^C]-methyl-L- and -D-tryptophan ([^11^C]-L-1MTrp and [^11^C]-D-1MTrp)	Small molecule	^11^C	Dynamic scan initiate during tracer infusion	–	Yes	(Xie et al. 2015)
IDO/TDO	L-5-[^18^F]fluoro-tryptophan and D-5-[^18^F]fluoro-tryptophan	Small molecule	^18^F	Dynamic scan initiate during tracer infusion	CT26, CT26-hIDO1, 17082A, 17095A	No	(Tang et al. 2017)
IDO/TDO	5-[^18^F]F-L-α-methyl tryptophan (5-[^18^F]F-AMT)	Small molecule	^18^F	30 min	B16F10	Yes	(Giglio et al. 2017)
IDO/TDO	1-(2-[18F]fluoroethyl)-l and d-tryptophan (1-L-[^18^F] FETrp and 1-D-[^18^F]FETrp)	Small molecule	^18^F	5 min, 2 h	Glioblastoma, NSCLC metastasis, breast cancer metastases, MDA-MB-231	Yes	(Michelhaugh et al. 2017; Xin and Cai 2017; Xin et al. 2019; Henrottin et al. 2016)
CD276	5573a	IgG	^89^Zr	1–7 d	MDA-MB-231	Yes	(Burvenich et al. 2018)
A2aR	[^18^F]FESCH and [^18^F]FPSCH	Small molecule	^18^F	Dynamic scan initiate during tracer infusion	Cerebral A2aR imaging	Yes	(Khanapur et al. 2017)
A2aR	[^11^C]KF17837	Small molecule	^11^C	Dynamic scan initiate during tracer infusion	Cerebral A2aR imaging, Myocardium	Yes	(Noguchi et al. 1998; Ishiwata et al. 2000a, 1997, 2000c; Alam et al. 2018)
A2aR	[^11^C]KF18446	Small molecule	^11^C	Dynamic scan initiate during tracer infusion	Cerebral A2aR imaging	Yes	(Ishiwata et al. 2000a, 2002, 2000c)

## Imaging the PD-1/PD-L1 axis

**Fig. 1:**
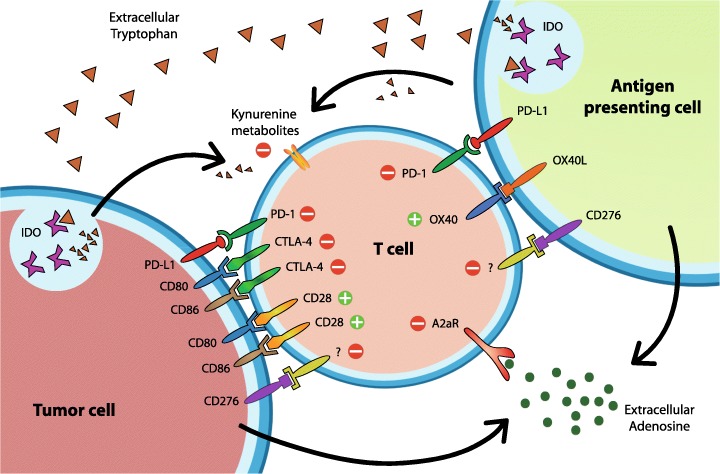
Immune checkpoint expression and main interactions on cell types which predominantly express them. Depicted are immune checkpoints for which tracers have been developed. Not all immune checkpoint interactions are known nor are all interactions displayed. For further reading on immune checkpoints, we refer to De Sousa Linhares et al. (2018) PD-L1: Programmed Death-ligand 1, PD-1: Programmed death-1, CTLA-4: Cytotoxic T lymphocyte associated antigen-4, A2aR: Adenosine 2a receptor, IDO: Indoleamine 2,3-dioxygenase

Programmed cell death 1 (PD-1 or CD279) is an immune checkpoint molecule present on T cells and immature B cells; its expression on CD8^+^ T cells is regulated by binding to the cognate T cell receptor (see Fig. 1). The continuous interaction between PD-1 and Programmed death-ligand 1 (PD-L1 or CD274) has been shown to induce and maintain metabolic exhaustion of lymphocytes (Sharpe and Pauken 2018), while PD-1 blockade re-invigorates exhausted CD8^+^ T cells; a concept that boosted further clinical development. PD-L1, one of the two known ligands for PD-1, protects healthy tissues against self-recognition by activated T cells. Cancer cells can exploit this immune suppressive mechanism to evade immune surveillance (Philips and Atkins 2015). In 2014, the first PD-1 Ab was approved by the FDA for advanced melanoma, and to date, more than 250 studies are actively investigating PD-1/PD-L1 based interventions (ClinicalTrials.gov 2019). These therapies have shown great promise and durable responses are increasingly observed. However, currently there is not a single biomarker that accurately predicts treatment response. PD-L1 status as determined on biopsied tumor tissue is only moderately correlated to treatment outcome and there is a need for more information regarding tumor status before and during immunotherapy (Shaverdian et al. 2017).

### PD-1 imaging

The potential of PD-1 imaging has been demonstrated in several preclinical and clinical studies. A copper-64 (^64^Cu) labeled anti-mouse PD-1 Ab was developed by Hettich et al. (Hettich et al. 2016). Studies in naïve and PD-1^−/−^ mice showed specific uptake in lymphoid organs (lymph nodes and spleen) of naïve mice, which was significantly lower in PD-1 knock-out mice at 24 h after injection, confirming the physiological expression of PD-1 in different immune compartments. In naïve mice with B16-F10 melanoma tumors that received PD-L1 and Cytotoxic T lymphocyte-associated protein 4 (CTLA-4 or CD152) directed ICI therapy, high tracer uptake was observed in the tumor upon irradiation, suggesting that PD-1 PET can be used for ICI treatment monitoring. Natarajan et al. injected a ^64^Cu-labeled anti-mouse PD-1 Ab in Foxp3^+^ LuciDTR4 mice, a mouse model that contains high expressing PD-1 Foxp3^+^ regulatory T cells, bearing B16-F10 tumors, to detect PD-1 expressing tumor infiltrating lymphocytes (TILs) (Natarajan et al. 2015). PET images obtained at 1–48 h after injection indicated tracer uptake mainly in the tumor and spleen, which was both found to be PD-1 specific T cell-mediated uptake confirmed by bioluminescence, IHC, and concurrent blocking of uptake by co-injection of an excess unlabeled tracer. In a subsequent study, Natarajan et al. developed a tracer against human PD-1. Pembrolizumab, a clinically approved anti-PD-1 Ab, was radiolabeled with ^64^Cu or zirconium-89 (^89^Zr) to detect tumor infiltration of adoptively transferred human peripheral blood mononuclear cells (hPBMCs) in NSG mice xenografted with human A-375 melanoma tumors (Natarajan et al. 2017). ^89^Zr-pembrolizumab uptake was observed in the tumor and spleen of hPBMCs engrafted mice at 24 h after injection. Specificity for PD-1 was demonstrated by the reduced ^64^Cu-pembrolizumab tumor uptake in mice coinjected with an excess of unlabeled pembrolizumab. In a final study by Natarajan et al., the ^64^Cu human PD-1 tracer was evaluated in NSG mice xenografted with 293 T/hPD-1 stable non-cancer cells and in NSG mice with adoptively transferred hPMBCs xenografted with A375 human melanoma cells (Natarajan et al. 2018a). PET imaging demonstrated specificity of the tracer towards PD-1 in 293 T/hPD-1 tumors in mice that did not receive an excess of unlabeled pembrolizumab compared to mice that did receive an excess of unlabeled tracer which showed significantly less tumor uptake. Moreover, ex vivo biodistribution indicated a statistically significant different tumor uptake between these two groups at 1, 24, and 48 h after injection. Studies with the adoptively transferred hPBMCs xenografted with A375 human melanoma mouse model indicated clear ^64^Cu-pembrolizumab uptake in tumors, suggesting infiltration of hPBMCs into the tumor microenvironment. Others evaluated the use of ^89^Zr-labeled nivolumab, a clinically used anti-human PD-1 Ab, in humanized (engrafted with peripheral blood mononuclear cells) or non-humanized NSG mice bearing subcutaneous A549 human lung tumors (England et al. 2018). PET imaging revealed higher ^89^Zr-nivolumab tumor uptake in humanized mice compared to non-humanized mice from 72 h onwards. Experiments comparing the uptake of non-specific ^89^Zr-IgG versus ^89^Zr-nivolumab in the humanized A549 tumor bearing mice confirmed the specificity of ^89^Zr-nivolumab tumor uptake. Interestingly, specific salivary gland uptake was observed that was mainly attributed to homing of lymphocytes due to graft versus host disease in this specific mouse model. These preclinical studies indicate that PD-1 imaging with PET might be a useful tool to image the presence of PD-1 expressing lymphocytes in the tumor microenvironment before ICI treatment or to image PD-1 expressing TILs during ICI therapy for treatment monitoring.

Recently, in a first-in-human clinical study by Niemeijer et al., ^89^Zr-nivolumab tracer uptake was evaluated in non-small cell lung cancer patients prior to nivolumab ICI treatment (Niemeijer et al. 2018). As expected from mouse models, tracer accumulation was observed in the spleen because of PD-1 expression on lymphocytes and Fc-receptor mediated uptake (Arlauckas et al. 2017) as well as in the liver because of tracer catabolism. More interestingly, a correlation between PD-1 expressing TILs by IHC in a primary tumor biopsy and ^89^Zr-nivolumab uptake was observed. Moreover, higher ^89^Zr-nivolumab uptake prior treatment was observed in responding tumor lesions than in non-responding tumor lesions after 3 months of nivolumab treatment. This study demonstrated that PET imaging can be used to quantify and monitor PD-1 expression non-invasively over time before ICI therapy.

### PD-L1 imaging

For PD-L1 imaging different tracer moieties have been explored, ranging from peptides, adnectins, up to full mAbs, labeled with various radionuclides for both PET and SPECT imaging of murine and human PD-L1 for mechanistic and translational purposes, respectively.

The first PD-L1 imaging agent was developed by Heskamp et al. who employed an ^111^In-labeled murine Ab directed against human PD-L1 and successfully imaged human xenografts in athymic mice with different PD-L1 expression levels (Heskamp et al. 2015). In a subsequent study, they investigated whether changes in PD-L1 expression on tumors could be visualized after radiotherapy using a mAb directed against murine PD-L1. Colon carcinoma (CT26) and Lewis lung carcinoma (LLC1) syngeneic mouse tumors showed significant increased tumor uptake after a single dose of 10 Gy external beam irradiation (Heskamp et al. 2019) which correlated to the increased IHC PD-L1 expression levels. Kikuchi et al. also investigated the effect of radiotherapy on the expression of PD-L1. A ^89^Zr-labeled mAb against mouse PD-L1 was used to show increased tracer uptake in a syngeneic head and neck tumor model after fractionated radiotherapy (Kikuchi et al. 2017). Other research teams have also successfully employed mAbs for imaging of PD-L1 using different radionuclides, including ^89^Zr, ^111^In, ^64^Cu and ^131^I (Hettich et al. 2016; Chatterjee et al. 2016; Josefsson et al. 2016; Nedrow et al. 2017a, 2017b; Lesniak et al. 2016; Li et al. 2018; Moroz et al. 2018; Truillet et al. 2018; Pang et al. 2018).

Next to mAbs, other moieties such as nanobodies, affibody molecules, adnectins and peptides have been tested in preclinical tumor models. Broos et al. developed a ^99m^Tc-labeled anti-mouse PD-L1 nanobody (also known as single domain antibody or VHH) (Broos et al. 2017). Experiments with immunocompetent mice bearing syngeneic myeloma TC-1 tumors showed specific physiological uptake in lungs, heart, spleen, thymus, lymph nodes and brown fat, as well as moderate tumor uptake. Others have shown successful studies using a ^64^Cu-labeled peptide that binds human and mouse PD-L1 and demonstrated high uptake in PD-L1^+^ xenograft models (Chatterjee et al. 2017). To achieve high-contrast images at earlier time points, they also optimized their peptide for a ^68^Ga-labeling. These results showed less tracer accumulation in xenograft tumors, but higher tumor-normal-tissue contrast in the PET images. However, high kidney and liver uptake was also observed (De Silva et al. 2018). Gonzalez et al. have shown results of a ^18^F-labeled affibody molecule which specifically targets human tumor PD-L1, but also demonstrates high renal and bone uptake (Gonzalez Trotter et al. 2017). Others have also published positive results with smaller targeting agents radiolabeled with ^99m^Tc, ^18^F, and ^68^Ga (De Silva et al. 2018; Donnelly et al. 2018; Ingram et al. 2017; Kumar et al. 2018; Mayer et al. 2017).

Niemeijer et al. performed the first in human PD-L1 imaging study with a PD-L1 targeting ^18^F-labeled adnectin (fibronectin binding domain 3 or monobody) (Niemeijer et al. 2018). Imaging with this low molecular weight tracer enabled same day imaging and illustrated the heterogeneous nature of PD-L1, both within and between patients with non-small-cell lung carcinoma (NSCLC). Most striking was the comparison of biopsied material against PD-L1 positive lesions on PET, where multiple cases of biopsy negative but scan-positive patients were observed. Furthermore, they showed therapy response was correlated with tracer uptake but not with biopsy findings. It must be noted that PD-L1 expression in these scan positive lesions was not confirmed with further biopsies and therefore we cannot conclude that these areas are indeed PD-L1 positive. However, these findings do suggest that PD-L1 can be expressed heterogeneously within and between tumors lesions and biopsies provide only limited information. Although the number of patients were limited, these results are encouraging for the future of immune checkpoint imaging in humans.

Bensch et al. performed patient imaging of PD-L1 with a clinically approved therapeutic mAb, ^89^Zr-labeled atezolizumab (anti-human PD-L1) (Bensch et al. 2018). Patients with metastatic bladder cancer, NSCLC or triple negative breast cancer being treated with atezolizumab were included. This study also found distinct heterogeneity of PD-L1 expression within and between patients on PD-L1 IHC. Furthermore, they showed a strong predictive value of PD-L1 PET imaging on progression-free survival as well as overall survival. When comparing PD-L1 PET to different PD-L1 IHC assays, they found that IHC could not predict treatment response and survival. This showcases the distinct value of imaging in patient stratification for immunotherapy, utilizing the therapeutic agent as a tracer.

Xing et al. evaluated a sdAb labeled with ^99m^Tc to visualize PD-L1 status in NSCLC patients on SPECT (Xing et al. 2019). They demonstrated safety and of their imaging compound and showed acceptable dosimetry when using ^99m^Tc. Furthermore, they were able to visualize PD-L1 positive tissues (spleen, liver and bone marrow) as well as tumors at 2 h post injection. As well as in the studies by Bensch and Niemeijer, heterogeneous uptake was found between primary tumors and nodal or bone metastases.

## Imaging CD28 and CTLA-4 and their ligands CD80 and CD86

The first molecule expressed by T cells in their activation cascade and required for their survival, is CD28, which binds both CD80 and CD86 present on antigen presenting cells (APCs). This stimulatory interaction can be inhibited by Cytotoxic T lymphocyte-associated antigen-4 (CTLA-4) which is also expressed by T cells and has a significantly higher affinity for CD80 (also B7–1) and CD86 (also B7–2) than CD28 (see Fig. 1). In a normal situation this inhibitory signal dampens T cell responses thereby avoiding collateral damage to healthy tissues (Acuto and Michel 2003; Zhao et al. 2018). So far several radiotracers for CTLA-4, CD80 and CD86 have been reported.

### CTLA-4 imaging

CTLA-4 is present on activated T cells and constitutively expressed by regulatory T cells as well as some types of tumor cells (Contardi et al. 2005). Counteracting the immune inhibitory effect of CTLA-4, the FDA-approved CTLA-4-inhibitor ipilimumab shows great anticancer efficacy in a wide range of cancer types. Despite the multitude of clinical therapeutical studies, more than 300 ongoing clinical studies for this mAb alone (ClinicalTrials.gov 2019), only four publications on imaging of CTLA-4 have been published. Higashikawa et al. (2014) used a ^64^Cu-labeled anti-murine CTLA-4 mAb to visualize CTLA-4 in CT26 tumor-bearing BALB/c mice. Tumor uptake was significantly higher in mice that received radiolabeled anti-CTLA-4 compared to mice that received radiolabeled non-specific IgG. Polymerase chain reaction (PCR)-analysis on CT26 tumor tissues from BALB/c and T cell lacking BALB/c nu/nu indicated that the CTLA-4 expression was T cell dependent and therefore the tracer could be used to image CTLA-4 positive T cells. In 2017, Ehlerding et al. reported uptake of ^64^Cu-labeled ipilimumab by CTLA-4 expressing human NSCLC xenografts (A549, H460, and H358) (Ehlerding et al. 2017). In vivo tumor tracer uptake correlated with in vitro CTLA-4 expression levels of these tumor cells, with the highest uptake in the A549 cell line 48 h post infusion. Furthermore, antigen specificity was evaluated by administration of excess unlabeled Ab to tumor bearing control mice. In a recent study, Ehlerding et al., validated the same anti-CTLA-4 tracer and a ^64^Cu-labeled IdeS protease fragmented ipilimumab F (ab’)_2_ in human peripheral blood lymphocytes engrafted NBSGW mice, a model that does not need full body irradiation to engraft human peripheral blood lymphocytes (PBLs) (Ehlerding et al. 2019). Both tracers showed targeting in salivary glands which upon IHC analysis showed activated CTLA-4 positive lymphocytes involved in a graft versus host disease. The F (ab’)_2_ tracer showed increased clearance, and thereby an increased salivary gland to blood ratios. Furthermore, tracer specificity was confirmed with non PBL engrafted NBSGW mice and radiolabeled nonspecific IgG isotype controls (Ehlerding et al. 2019). Ingram et al. developed H11, an anti-CTLA-4 VHH that can be ^18^F or ^89^Zr-functionalized (Ingram et al. 2018). In vivo targeting of ^18^F-H11 in mice bearing T cell containing B16F10 tumors showed tracer uptake above background. A ^89^Zr-labeled 20 kD PEG conjugated H11 (H11PEG20) showed a substantially improved signal to noise ratio in the tumor and uptake in the GVAX injection site, a tumor model specific immune stimulatory cancer vaccine that was applied before tumor inoculation, indicating activated T cell specific targeting. These results indicate the feasibility of using radiolabeled anti-CTLA-4 agents for assessment of TIL or tumor CTLA-4 expression levels. Currently, a first clinical CTLA-4 imaging study is ongoing, where tumor lesion uptake and biodistribution of ^89^Zr-labeled ipilimumab will be assessed at the start of ipilimumab therapy and 3 weeks post start of therapy. Furthermore, this study is designed to determine a possible correlation between tumor uptake and therapy responses, uptake in normal tissues and to assess a correlation between ‘on-target off-tumor’ targeting and toxicity (Philips and Atkins 2015).

### CD80 and CD86 imaging

CD80 and CD86 are expressed mainly by APCs and their binding to CD28 and CTLA-4 stimulates and inhibits immune responses, respectively. These molecules are also expressed by some types of myelomas, lymphomas and carcinomas (Flörcken AaJ et al. 2017). Conditionally being either immune stimulatory or inhibitory, imaging CD80 or CD86 expression in tumors might be used to predict response to CTLA-4 targeted therapies. Alternatively, in case of CD80/CD86 negative tumor cells, imaging of these targets could be used to non-invasively measure APC infiltration. Furthermore, if the ongoing therapeutical clinical trial with CD80/CD86 targeting CAR-T cells turns out successful, nuclear imaging could aid in patient selection for this treatment approach (ClinicalTrials.gov 2019). Meletta et al. investigated in vivo tumor targeting of ^111^In-labeled belatacept (Meletta et al. 2016). This tracer, a fusion protein consisting of a human IgG1 Fc fragment linked to the extracellular domain of CTLA-4, showed higher uptake in CD80^+^/CD86^+^ Raji tumors (a Burkitt’s lymphoma) compared to uptake in double negative NCI-H69 tumors. Furthermore, co-injection with excess unlabeled belatacept in Raji tumor-bearing mice showed significantly decreased tracer uptake, indicating specific receptor targeting of the tracer. Furthermore, Meletta et al. have developed a ^11^C-labeled pyrazolocinnoline derivative AM7 to target CD80 positive cells (Meletta et al. 2017). In vivo uptake of this tracer was low in atherosclerotic plaques rich with CD80 expressing macrophages compared to [^18^F]FDG. Tracer specificity for its target was confirmed by in vitro autoradiography and IHC (Muller et al. 2014). Although no studies have been performed in patients, these studies suggest the feasibility of assessing CD80 and CD86 expression with radiolabeled tracers.

## Imaging other immune checkpoint molecules

### IDO and TDO imaging

Decreased extracellular tryptophan levels and increased kynurenines levels (tryptophan metabolites) inhibit T and NK cell proliferation and activation, therefore tryptophan is a metabolic immune checkpoint (see Fig. 1). Some tumors exploit this immune modulating mechanism by over-expressing tryptophan degrading enzymes indoleamine 2,3-dioxygenase (IDO1 and IDO2) and tryptophan 2,3-dioxygenase (TDO) (Platten et al. 2014; Munn and Mellor 2007). Several inhibitors for the rate limiting enzyme IDO1 (such as Epacadostat, Indoximod and Navoximod) have been developed and are currently undergoing evaluation in clinical trials as adjuvants (Prendergast et al. 2017).

Multiple tracers for assessing expression levels of IDO1/TDO have been developed. All tracers, except an ^18^F-labeled epacadostat analog developed by Huang et al. (2017), are ^18^F or ^11^C-labeled derivatives of either L or D isomers of tryptophan (Table 1). Although already developed in 1988, the first clinical study with the IDO1 tracer α-[^11^C]methyl-L-tryptophan ([^11^C]AMT) was performed in 2006. In patients with brain tumors, [^11^C]AMT-PET demonstrated increased tracer uptake in the tumor compared to normal cortex (Juhasz et al. 2006). Moreover, multiple clinical [^11^C]AMT-PET trials demonstrated prolonged tracer retention and high uptake in NSCLC lesions (Juhasz et al. 2009), invasive ductal breast carcinoma lesions Juhasz et al. (2012) and meningiomas Zitron et al. (2013). Further clinical studies with [^11^C]AMT-PET demonstrated the ability to differentiate radiation necrosis from recurrent gliomas (Alkonyi et al. 2012), and showed a strong association of high tumor [^11^C] AMT uptake parameters (SUVmax, SUVmean and tumor-to-background ratio) to a significantly decreased 1-year survival (Kamson et al. 2014). Although a recent clinical [^11^C]AMT-PET study in 3 patients failed to demonstrate objective clinical responses to IDO inhibitor therapy, it did show heterogeneous intratumoral tracer uptake potentially reflecting IDO activity (Lukas et al. 2019). Furthermore, these results indicate that [^11^C]AMT-PET might be used for stratification of true progression versus pseudoprogression. In a recent preclinical study, in vivo tumor targeting of a newly developed IDO1 tracer, 1-(2-[^18^F]-fluoroethyl)-L-tryptophan ([^18^F]FETrp), was compared with [^11^C] AMT and increased SUVs were observed for [^18^F] FETrp compared with [^11^C] AMT in lung, breast, and brain xenografts (Michelhaugh et al. 2017). Target specificity of [^18^F] FETrp has also been demonstrated in preclinical prostate, lung, breast, and glioma tumor models by Xin et al. with a significantly higher tumor uptake than in low IDO1 expressing healthy tissues, and a correlation of in vitro cell binding and in vivo [^18^F] FETrp tumor uptake. So far only [^11^C] AMT imaging agent is being investigated clinically to evaluate whether IDO1 imaging could serve as a predictive marker for immunotherapy. This study, [^18^F] FDG PET 48 h before pembrolizumab and [^11^C] AMT imaging 24 h before pembrolizumab, will investigate a possible association of the SUVmax with objective PD-1 between tracer uptake, PD-1 inhibitor therapy response and IHC (Philips and Atkins 2015).

### CD276 imaging

CD276 (also known as B7-H3) is presented at low levels on healthy tissues but highly expressed by APCs and macrophages and by a range of solid tumors. Although its target is still unknown, ample evidence is present that show its involvement in inhibiting T cell function (see Fig. 1) (Dong et al. 2018). This is substantiated by the antitumor effects of currently preclinically and clinically investigated CD276 inhibitors or targeted therapies (ClinicalTrials.gov 2019; Lee et al. 2017). To aid patient selection for CD276 inhibitor therapy, tracers are being developed. A humanized anti-B7-H3 mAb, ^89^Zr-labeled 5573a, has been used in immunodeficient CD276^+^ MDA-MB-231 tumor-bearing mice for non-invasive imaging of CD276 (Burvenich et al. 2018). PET/MRI and biodistribution analysis showed significantly higher tumor uptake compared with mice that were co-injected with an excess unlabeled tracer, indicating CD276 specificity. This tracer demonstrates the potential of in vivo CD276 imaging. However, further preclinical evaluation in immunocompetent mice is warranted in order to better understand tracer behavior and to translate findings to patients.

### A2aR imaging

The metabolic inhibitory immune checkpoint Adenosine 2a receptor (A2aR) is expressed by neurological synapses, certain tumor cells, and a wide range of immune cells (e.g. macrophages, T cells and monocytes). Binding of adenosine to tumor-expressed A2aR promotes tumor cell proliferation and metastasis, whereas ligation of immune cell-expressed A2aR suppresses immune function (see Fig. 1) (Sek et al. 2018). Together with increased extracellular adenosine levels caused by inefficient ATP production by tumor cells, a wide range of tumors exploit this pro tumorigenic and immune suppressive mechanism by expressing extracellular adenosine level increasing enzymes (CD39 and CD73) (Gao et al. 2014). Various A2aR and CD39/CD73 antagonistic therapies have been developed and show encouraging preclinical anti-tumor results warranting the multiple ongoing clinical studies (ClinicalTrials.gov 2019). Noninvasive nuclear imaging could aid in the evaluation of new antagonists and patient selection for A2aR and CD39/CD73 therapy. So far, all imaging studies have focused on A2aR, probably because adenosine targeting tracers risk altering the autoimmunity preventive effects of circulating adenosine. Furthermore, high adenosine levels in plasma could also interfere with targeting of adenosine in the tumor. Papers describing preclinical and clinical A2aR imaging studies have shown impressive results for [^11^C] Preladenant, [^11^C] TMSX and ^18^F-labeled SCH442416-analogs in a number of applications, most of which for intracranial A2aR imaging (Noguchi et al. 1998; Zhou et al. 2017a, 2017b, 2017c, 2017d; Rissanen et al. 2013; Ramlackhansingh et al. 2011; Sakata et al. 2017; Mishina et al. 2007, 2011; Naganawa et al. 2007, 2014; Lahesmaa et al. 2018; Khanapur et al. 2014, 2017; Ishibashi et al. 2018; Ishiwata et al. 2000a, 2002, 2000b, 1997, 2000c). No studies with these tracers have yet been performed to asses tumor expression of A2aR, but as some have already demonstrated to be safe for in human use, translation on short term to the field of oncology should be achievable.

### OX40 imaging

Binding of the ‘second wave’ co-stimulatory receptor OX40 (also known as CD134) to its ligand OX40L promotes T cell activation (see Fig. 1). Several agonistic biologicals for this stimulatory immune checkpoint have already shown objective responses in phase I clinical trials as mono or combination therapy (Infante et al. 2016; El-Khoueiry et al. 2017). To complement these therapies, nuclear imaging of the T cell activation marker OX40 might be used to predict OX40 agonist responses or to follow treatment responses that focus on T cell activation. One study describing the development and in vivo characterization of a tracer binding OX40 has been published. In 2018, Alam et al. showed that the ^64^Cu-labeled murine Ab AbOX40 could be used to image OX40 noninvasively and longitudinally (Alam et al. 2018). In this study, dual A20 lymphoma-bearing mice received either an immune stimulant (Cytosine phosphodiester Guanine-oligodeoxynucleotides (CpG-ODN), microbial signature DNA fragments) or vehicle only intratumorally in one tumor, the second tumor was untreated. Two days post treatment, PET imaging demonstrated increased ^64^Cu-AbOX40 uptake in CpG-ODN treated tumors and their draining lymph nodes compared to vehicle treated and untreated tumors. Furthermore, a notable increased tracer uptake was observed in the tumor draining lymph nodes and spleen 9 days after treatment. These findings demonstrate that anti-OX40 Abs are suitable for in vivo PET imaging of activated T cells.

## Further targets of interest

Besides the targets mentioned above, we believe there are other immune checkpoints which are potentially important for future immune checkpoint inhibition therapies and could become important targets for imaging as well. For example, LAG-3 and TIM-3 play a major role in the activation, proliferation and homeostasis of T cells. Multiple clinical trials are currently ongoing to evaluate the safety and efficacy of pharmaceuticals targeting LAG-3 and TIM-3 and a preclinical study is investigating potential of an imaging tracer directed against LAG-3 (Vivier et al. 2019). Another target of interest is V-domain Ig suppressor of T cell activation (VISTA). Antagonists blocking its inhibitory functions, resulted in increased immune activation in multiple mouse models. Expression of VISTA in immune checkpoint blockade therapy resistant patients could open up alternative treatment options. Finally, preclinical studies have demonstrated that Inducible T cell costimulatory (ICOS or CD278) agonistic therapy improve the efficacy of other immune checkpoint therapies, and imaging could therefore be of interest to predict response or stratify patients for combination therapy.

## Discussion

Current studies demonstrate the wide variety of successful immune checkpoint imaging approaches. As is the case with all radiotracers, high target affinity, stable in vivo behavior, and adequate specificity with minimal uptake in target negative tissue are desired when performing imaging studies. As opposed to imaging of (often) highly upregulated tumor related targets, immune checkpoints are mostly expressed by highly mobile immune cells, or in some cases at physiological levels by tumor cells, and can be dynamic in their expression levels over time. Because of this, determining optimal imaging timepoints during the course of disease is more critical than in imaging studies exploiting the constitutively highly expressed tumor targets. Also, available immune imaging targets in tumor lesions can be low compared to physiological expression levels in other immune-related organs in the body e.g. the spleen. Finally, because of the inherent immunological functions of immune checkpoints, there is a need to verify the effects tracers can exert on their target cell population; e.g. ‘on-target off-tumor’ immunogenicity or target cell differentiation or even depletion. Thus, to develop tracers for immune checkpoint imaging with favorable characteristics some issues require special attention.

First, determining in vivo targeting specificity is vital. To asses specificity, blocking studies can be performed. Here, the addition of an excess unlabeled tracer is used to block the labeled tracer from binding to its target in a concentration dependent manner, remaining tracer uptake must then be attributed to aspecific EPR effects. However, this method does not take into consideration dose-dependent biodistribution effects (for instance: sink organs) and specific Fc-mediated uptake, which may also reduce upon injection of an excess of unlabeled antibody. Alternatively, knockout mice can be used; by missing the target protein of interest all eventual uptake can be considered as non-target specific. The limitation here is that due to the genetic modification, developmental changes may occur and the resulting animal might not be sufficiently comparable to the baseline strain. Finally, to asses aspecific uptake it is also possible to use scrambled peptides, isotype control antibodies or other relevant controls depending on tracer moiety.

Second, because of their physiological role in regulating the immune response, immune checkpoints can be expressed by a wide variety of tissues, including cancer cells, subsets of immune cells, but also non-lymphoid tissues such as activated endothelial cells, brown fat, or duodenum (Heskamp et al. 2019). The physiological target expression throughout the body leads to ‘on-target-off-tumor’ distribution of tracer. Moreover, as with PD-1, the same immune checkpoint can be expressed by immune cells with immune stimulatory, suppressive or effector functions. Alternatively, certain immune checkpoints, like CD80 and CD86, can both stimulate or inhibit immune responses. Therefore, choosing the animal model and conditions that will yield relevant results is essential. Knowledge of the expression levels on different immune cells and their location is not only essential in order to accurately interpret the acquired PET or SPECT image, but also to find optimal dosing levels. For example, PD-L1 is highly expressed by splenic cells and therefore the spleen acts as a sink organ. As a consequence, at low tracer doses, all injected tracer will accumulate in the spleen, resulting in rapid blood clearance and minimal targeting to other PD-L1 positive tissues like the tumor (Heskamp et al. 2019; Nedrow et al. 2017b). By increasing the tracer dose, spleen uptake can be saturated, resulting in restored circulation time and increased targeting to tumor and other PD-L1 positive tissue. However, this is not the case for all immune checkpoints or their ligands. For example, expression levels of PD-1 are much lower and there is no sink organ affecting the biodistribution of PD-1 targeting tracers, therefore lower doses should be used to prevent saturation of all PD-1 and to obtain high contrast images (Hettich et al. 2016).

Third, when targeting immune checkpoints expressed on immune cells, the number of immune cells in a tumor may be below detection limit. Therefore, in order to be able to detect these low number of cells, it is essential that the tracer demonstrates high and specific uptake and can be labeled with sufficiently high specific activity. IgG based imaging moieties will result in high absolute uptake in tissue of interest but also a long circulation time, leading to imaging timepoints of 24 h and upward with low signal to background contrast at early time points post injection. They are however widely available and can be easily coupled with various SPECT and PET isotopes. The presence of the Fc region in antibodies has a large influence on its in vivo distribution, for example Fc-mediated recycling and degradation by endothelial and immune cells leads to a long circulation time and high liver uptake. Furthermore, antibodies are potentially immunogenic, depending on the antibody isotype. This can cause anti-target immune activation, complement activation or the formation of anti-drug antibodies, which could lead to target cell depletion, altered tracer pharmacokinetics and serious adverse events when there is cross-recognition of normal proteins; also it limits repetitive imaging in animals. To prevent this, the glycosylation in the Fc domain can be modified, resulting in reduced FcRy mediated uptake (Vivier et al. 2019). Despite these drawbacks, many therapeutic immune checkpoint targeting agents are antibodies and by radiolabeling these agents, they can be used for theranostic purposes. Thus, molecular imaging with therapeutic antibodies is still of great value. However, the use of smaller tracers (nanobodies, minibodies, affibodies, peptides, adnectins etc.) does have some advantages over intact antibodies. For example, it can result in higher target-to-background signals. Because of the rapid pharmacokinetics of smaller tracers, imaging is usually possible within 30 min up to a few hours post injection, while lowering radiation exposure to the patient. Due to the absence of the Fc-region, there is no Fc-mediated (re-)activity. However, these molecules need to be optimized in terms of affinity and specificity in order to result in favorable in vivo behavior. The optimum combination of different conjugations, PEGylation, spacers, chelator combinations, and an isotope matched to the biological half-life of the tracer is paramount to develop a good imaging moiety. PET may also be the preferred imaging method over SPECT because of the higher sensitivity and resolution, especially in the clinical setting. Although in the preclinical setting, SPECT has a higher resolution which can be used to study the heterogeneity within a tumor in more detail.

Fourth, immune checkpoint-targeting radiotracers should be carefully designed so that they do not interfere with nor alter normal functioning of target immune cells. For example, upon binding of the tracer, irradiation might damage the immune cells and different subsets of immune cells demonstrate differences in radio sensitivity. Potentially this could alter their function, although literature reports T cell labeling with ^89^Zr up to 0.5 MBq/10^6^ cells without negative effect on viability over a period of 7 days (Bansal et al. 2015; Charoenphun et al. 2015). Moreover, terminally differentiated effector cells have a short biological lifespan. Furthermore, high doses of immune-cell targeting Abs might result in Fc receptor mediated depletion of these immune cells. This can be circumvented by using micro doses of Ab, or by developing tracers with a modified Fc domain, or lacking an Fc domain (Tavare et al. 2014, 2015).

Finally, in the preclinical setting it is essential to select the right animal model. It is known that different immunocompetent mouse strains show varying immune responses when encountering the same antigen. Many radiotracers described in literature have been tested in immunodeficient mice engrafted with human tumors, and often the radiotracers do not cross react with the murine immune checkpoint. Although this allows a first characterization of the radiotracers, it might be difficult to translate the findings to the clinical setting as immune checkpoints are also expressed on normal tissues. The use of humanized mice (mice transplanted with human immune cells) will overcome some of the limitations. However, these animals still do not have a fully functioning immune system, for example they lack mature T cells, or when T cells are present, graft-versus-host responses might occur. This can be used to collect additional proof of target specificity as observed by Ehlerding et al. in their CTLA-4 imaging studies. However, due to the inherent instability and ultimately lethal complications of such a transplanted foreign immune system, longitudinal studies are challenging. Finally, although these humanized mice develop human immune cells, the other tissues are still completely murine, therefore these models are not relevant for determining pharmacokinetics, biodistribution, immunogenicity or depletion effects. Therefore, many researchers make use of tracers specifically developed to detect murine immune checkpoints as this allows for experiments in animals with a fully functioning immune system.

### Future prospects

To realize the full potential of immune checkpoint imaging, it is essential that novel immune checkpoint tracers are developed according to the requirements that serve their (pre) clinical application. The first step to achieve this is to select the optimal tracer for preclinical or clinical research, keeping in mind the specific criteria discussed in the previous section. Once these tracers have been validated, they can play an important role in early drug development, as it can provide information about pharmacokinetics, tumor targeting, and potential off-tumor accumulation which could lead to adverse side-effects of immune checkpoint inhibitors. Longitudinal molecular imaging will also aid in elucidating dynamic expression and interactions of immune checkpoints, and thus aid in better understanding of tumor immunology and providing new insights for therapeutic interventions. When a novel ICI has shown promising anti-tumor effects, imaging can be used to facilitate fast acquisition of preclinical and clinical experimental data on multiple treatment combinations to ultimately design the most rational treatment plan for a specific tumor immunological phenotype. Correct analysis of acquired images hinges on understanding the immune checkpoint expression in relation to therapy. As already shown by Niemijer et al., patient stratification based on whole-body PET imaging is a viable tool to facilitate treatment individualization (Niemeijer et al. 2018). However, in order to achieve this in clinical practice, high sensitivity and specificity, as well as access to practical preparation of the radiopharmaceutical are necessary.

Given the wealth of unique data that can be derived from in vivo checkpoint imaging prior to and during novel immune therapeutic (combination) strategies, complementary to tissue sampling; it should be stimulated to apply molecular imaging tools in early stages of drug development. Collaborative approaches between pharmaceutical industry and academic partners have shown their impact in studies using currently approved radiolabeled checkpoint inhibitors; and it is envisioned that this fosters similar studies with next generation of immune therapies. In particular, when new clinical trials combining different immune checkpoint therapies are being initiated. With only limited number of patients available to include in these trials (Tang et al. 2018), strict patient screening for treatment eligibility should be applied.

## Conclusion

The introduction of immune checkpoint therapies initiated a new era of effective immune therapy. The decision to treat patients with immune checkpoint therapies currently depends on immune checkpoint expression and infiltration of immune cells detected with IHC, which requires invasive biopsies. In this review we have discussed the potential of molecular imaging for immune checkpoint therapy drug development, patient selection, and therapy individualization. For PD-L1 and PD-1, the strength of PET imaging in immunotherapy was recently underlined by first-in-human trials correlating uptake of immune checkpoint tracers with immunotherapy outcome (Niemeijer et al. 2018; Bensch et al. 2018). Since these studies have shown that PET imaging of immune checkpoint expression in tumor lesions is safe and feasible, the road is open for future clinical trials to validate the use of PET as a complementary diagnostic tool to IHC for patient stratification prior to ICI therapy, for treatment monitoring during therapy, and as a tool in early cancer drug development (Graphical abstract). Ultimately, imaging with these new immune checkpoint tracers could aid to speed up development and implementation of effective mono- and combination therapies, treatment of only patients that potentially benefit and preventing severe side-effects in patients that will not benefit from ICI therapy.

## Data Availability

Not applicable

## Abbreviations

A2aR: Adenosine 2a receptor
CD8/28/80/86/276/279: Cluster of Differentiation
CTLA-4: Cytotoxic T lymphocyte associated antigen 4
FDA: Food and Drug Administration (U.S.)
FDG: Fludeoxyglucose
Foxp3: Forkhead box p3
hPBMC: Human peripheral blood mononuclear cell
ICI: Immune checkpoint inhibition
IDO1: Indoleamine-pyrrole 2,3-dioxygenase 1
IHC: Immunohistochemistry
mAb: Monoclonal antibody
MRI: Magnetic resonance imaging
NBSGW: NOD.Cg-Kit^W-41J^ Tyr^+^ Prkdc^scid^ Il2rg^tm1Wjl^/ThomJ
NSCLC: Non small-cell lung carcinoma
NSG: NOD scid gamma
PBL: Peripheral blood lymphocyte
PD-1: Programmed cell Death 1
PD-L1: Programmed cell Death Ligand 1
PET: Positron emission tomography
sdAb: Single domain antibody
SPECT: Single photon emission computed tomography
SUV: Standardized uptake value
TIL: Tumor infiltrating lymphocyte
